# Contribution to the Knowledge of Flesh Flies (Diptera: Sarcophagidae) Attracted to Baits of Decomposing Animal Tissue in Athens, Greece: Ecological Patterns with Potential Forensic Entomology Applications

**DOI:** 10.3390/insects17101000

**Published:** 2026-09-27

**Authors:** Gabriella Dimitra Rakopoulou, Sotiris Alexiou, Rebecca Baldwin, Georgette Kluiters, Ian R. Dadour

**Affiliations:** 1 Entomology and Nematology Department, University of Florida, Gainesville, FL 32611, USA; baldwinr@ufl.edu (R.B.); g.kluiters@ufl.edu (G.K.); 2 Korinthian Museum of Natural History, Isthmion 201, 20100 Korinthos, Greece; sotirisalexiou@hotmail.com; 3 School of Medical, Molecular & Forensic Sciences, Murdoch University, Murdoch, WA 6150, Australia; mygalopsis@gmail.com; 4 School of Medical and Health Sciences, Edith Cowan University, Joondalup, WA 6027, Australia

**Keywords:** Diptera, *Sarcophaga*, forensic entomology, carrion baited traps, species diversity, insect ecology

## Abstract

The current study details the first species list of flesh flies (Diptera: Sarcophagidae), collected using carrion baited traps at three locations within Athens, Greece. The research aims to understand the local flesh fly fauna and discuss their potential use in forensic applications. Employing traps baited with beef liver, fish, and chicken stomach, the present study assessed the efficacy of these different bait types in attracting Sarcophagidae. The study elucidates species diversity, richness, species assemblage, abundance, and bait preference. A total of 1125 *Sarcophaga* spp. specimens were captured, of which 241 were males, representing 12 species. The predominant species were *Sarcophaga africa* (Wiedemann) and *Sarcophaga melanura* Meigen. Overall, among the identified males, beef liver attracted the highest number and greatest species diversity of flesh flies, followed by fish and finally chicken stomach. Identification of this insect group contributes to the discipline of Forensic Entomology in Greece by providing a comprehensive database of local flesh fly species associated with carrion that may provide information about the circumstances concerning the time since death (TSD) in crime scene investigations.

## Simple Summary

Flesh flies are an important group of insects in forensic science because their presence on decomposing remains can aid in the estimation of the time since death (TSD). However, information on this group and its association with carrion in Greece has been limited. The present study provides the first species list of flesh flies collected with carrion baited traps at three locations in Athens. Traps baited with beef liver, fish, and chicken stomach were used to examine species diversity, abundance, and bait preference. A total of 1125 *Sarcophaga* spp. specimens were captured, of which 241 were males, representing 12 species. The most abundant species were *Sarcophaga africa* (Wiedemann) and *Sarcophaga melanura* Meigen. Beef liver attracted the highest number of flies and the greatest species diversity, followed by fish and chicken stomach. Ecologically, these findings provide valuable data on local flesh fly communities in Athens and may support future forensic investigations in Greece by improving the interpretation of insect evidence recovered from decomposing remains, particularly when estimating TSD.

## 1. Introduction

Forensic Entomology (FE), the use of insects and other arthropods in criminal and legal investigations [1], relies on the interpretation of necrophagous insects collected from decomposed remains [2,3]. Adult flies are typically the first insects to arrive at a fresh carcass, capable of detecting and locating remains within hours, minutes [4], and sometimes seconds [5], following death. FE primarily provides information concerning the minimum postmortem interval (PMImin) [3,6,7], a process that involves interpreting the development of immature stages of necrophagous insects colonizing remains and their association with the decomposition process [8].

The flesh flies (Diptera: Sarcophagidae) are the second-largest family within Oestroidea [9] and are acknowledged as one of the most abundant and ecologically diverse insect groups exploiting ephemeral resources [10]. The group is speciose, comprising approximately 3000 species across more than 100 genera, with ~900 species classified within the genus *Sarcophaga* Meigen [11,12,13]. Flesh flies are ubiquitous in subtropical and tropical synanthropic environments [2,14], and, although less prevalent, they have been recorded in temperate climates [15,16]. Sarcophagids are typically ovoviviparous, depositing live larvae directly onto animal remains, while a few species are oviparous [2,17,18,19,20]. Adult flesh flies are regular visitors of carcasses, exploiting both small and large carrion, including human remains [16]. They have been noted visiting decomposing carcasses outdoors and indoors, in sunlit or shaded sites, as well as across a spectrum of climatic conditions, ranging from wet to dry environments [21]. Sarcophagids typically occur during the early stages of decomposition; however, they have been recorded during the later stages of this process [2,16,22]. Consequently, this taxon is of considerable forensic importance [16].

Flesh flies, notwithstanding their medicolegal value, have remained underutilized in forensic investigations [16,23]. Although some aspects of sarcophagid biology and ecology still remain insufficiently understood [2], European field studies have provided important insights into their role in carrion communities [16]. This understanding is crucial for determining the forensic relevance of a given taxon. Although less known and studied compared to other necrophagous flies [e.g., blowflies (Diptera: Calliphoridae)], their relevance for forensic purposes has been increasingly recognized and discussed in numerous publications [16,24,25,26,27].

There are two primary factors that contribute to the reluctance of forensic entomologists using flesh fly taxa for PMImin estimations [12]. First, systematic studies on both immature and adult taxonomic characters remain inadequate [2,12,28]. Globally, a major challenge arises from the morphological similarities among females, both intra- and interspecific [12]. In Europe, however, identification has been facilitated by the availability of regional studies [16]. The second factor is mutually inclusive of the first factor, as insufficient documentation exists regarding their thermo–biological life histories, habits, and behaviors [2,12]. In order to characterize the diversity of an insect group, local databases should incorporate as many taxa within the target group as possible [2,4,29]. Locality specific data are valuable in all areas where FE is utilized, to provide baseline data for comparisons with taxa collected at crime scenes [30].

The use of traps baited with carrion is a widely adopted technique for saprosarcophagous species, with numerous studies utilizing various trap and bait types to assess species attractiveness and preferences across different natural habitats [4,29,30,31,32,33]. Baited traps are inexpensive, have no chemical agents, do not harm the environment, can be deployed in accessible locations, and are generally not disturbed by scavengers [29,30,33]. Regional and local studies using bait trapping have contributed to the development of faunistic checklists, the understanding of factors affecting saprosarcophagous species diversity and the dynamics of local populations [29,34,35,36,37,38].

Despite their medical [39], veterinary [40], and forensic relevance [41], the current state of knowledge concerning Sarcophagidae remains deficient in many regions [2,12,42,43], including Greece [37,44]. Until recently, knowledge of the flesh fly fauna of Greece remained fragmentary, with most species recorded from other Mediterranean regions [45,46]. This has changed substantially due to recent contributions by Zafeiriou et al. [37] and Rakopoulou et al. [38]. These new records from Greece provide a basis for advancing systematic studies and establishing a framework for future faunistic, ecological, and forensic research on this taxonomic group.

In this context, the principal objective of this study is to provide information on the faunal composition and ecological patterns of flesh flies occurring in Athens, Greece. To our knowledge, within Greece, this is the only study of the family Sarcophagidae carried out exclusively in an urban environment using three distinct decomposing baits of animal origin. This study aimed to elucidate sarcophagid diversity, richness, species composition, abundance, and bait attractiveness. The data collected on local flesh fly fauna have the potential to deliver information regarding the circumstances surrounding TSD from animal remains in forensic investigations and support the integration of FE into the Greek judicial system.

## 2. Materials and Methods

### 2.1. Study Area

An experiment was conducted in three locations within the urban area of Votanikos [Agricultural University of Athens (AUA)], Athens, Attiki, Greece. According to the Koppen–Geiger Climate Classification, Athens experiences a Csa—Hot Mediterranean climate characterized by prolonged hot, dry summers and mild, wet winters [47]. The urban environment surrounding the AUA is characterized by intense commercial activity and environmental degradation. The numerous experimental plant plots within the AUA include livestock breeding areas, aquaculture facilities, greenhouses, beekeeping and sericulture units, a vineyard, an arboretum and a botanical garden. To obtain representative samples of the flesh fly species inhabiting this location, three sites within the AUA were selected for this study. The three sampling locations are depicted in Figure 1.

The first site was in the experimental orchard (37°58′53.2″ N, 23°42′21.6″ E), which includes a variety of cultivated tree species (citrus, pistachio, figs, stone fruits, olive, carob) covering a total area of 5000 m^2^. The second site was near the experimental livestock facilities (37°59′05.3″ N, 23°42′22.6″ E), where numerous cattle, sheep, pigs, and poultry are housed alongside their waste storage areas. The third site was located within the arable crops area (37°59′02.9″ N, 23°42′10.4″ E), which includes various grains, legumes, and other crops such as *Cannabis sativa* L. and *Helianthus annuus* L. No insecticides or herbicides were applied at any of these locations during the study period. Weather conditions in the field, including the average temperature and relative humidity, were monitored throughout the study using a weather station (Model: Davis Vantage Pro 2—Automatic Weather Station) located within the AUA.

### 2.2. Trapping Methodology

Two trap types were employed to catch fly specimens: bottle traps and pitfall traps. Three bottle traps and three pitfall traps were placed at each of the three locations, a total of 18 traps. All baits were purchased from a meat vendor and were free of chemicals. Each trap contained one type of animal tissue: fresh beef liver, chicken stomach, or fish (Figure 2). For each trap type at each site, one trap was baited with 50 g of fresh chicken stomach, one with 50 g of fresh beef liver, and one with 50–60 g of whole fresh sardine, resulting in six traps per bait type across the three sites. Baits were supported on a metal hook attached to the lid of each trap.

All traps were refurbished plastic containers with two 1 cm diameter openings to release the odor of the attractant into the surrounding environment and allow insect entry. Bottle traps were hung from tree branches approximately 1.5 m above the ground for convenient operation and effectiveness [48] and to reduce the risk of vertebrate scavenging. On the other hand, pitfall traps, while being susceptible to vertebrate scavenging, were available to walking insects and were buried in the ground, with entry access to insects at the soil surface. Each trap contained 200 mL of deodorized 95% ethyl alcohol as the insect preservation fluid. The distance between each of the three sites was approximately 500 m. The six traps (three bottle and three pitfall traps) at each locality were separated by at least 5 m. Traps were sampled a total of 27 times during the study period (May–November 2021), and there was no evidence of any trap damage due to scavengers. During each sampling event, trap openings were blocked to prevent any insects from escaping. Once all the insects were extracted from each trap, the metal hooks were disinfected with bleach, washed with H_2_O, allowed to dry, and replaced with fresh baits. Traps were topped up with deodorized 95% ethyl alcohol. All adult insects were washed with H_2_O and 95% ethyl alcohol, dried on absorbent paper, and pinned. The time taken for trap replacement was 30 min. All sarcophagid species captured in either trap at each site were combined for analysis. Other captured insects were excluded from the current analyses.

### 2.3. Identification of Specimens

After extraction from the traps, all insect specimens were pinned, enumerated, and sexed. All female sarcophagid specimens were identified to the genus level. Taxonomic identifications were based on external morphology. Only male specimens were identified to species level by dissection followed by an examination of the male aedeageal structures. Male terminalia were exposed using entomological tweezers, followed by placing the genitalia into a 10% solution of potassium hydroxide (KOH), making the soft tissue translucent. To validate the identification of each specimen, taxonomic characters of each species were examined using a stereoscopic microscope (Model: BMS 133 Zoom Trino, LED), alongside specific taxonomic revisions, descriptions, specialized taxonomic keys, and other available publications [45,49,50,51,52,53,54]. Post identification, genital structures were preserved in glycerin solution and stored in plastic microvials. A voucher collection of the identified adult male specimens of the present research was consigned to the entomological collection of the Korinthian Museum of Natural History (KMNH), Korinthos, Greece.

### 2.4. Data Analyses

The number of sarcophagid taxa captured in each trap type at each site was combined for each sampling event, and these data were used in the ensuing analyses. Trap captures were pooled for analysis because the study focused on overall male sarcophagid diversity and bait attractiveness rather than trap type comparisons. The data were evaluated using defined “Dominance” and “Frequency” criteria, as demonstrated in Rakopoulou and Dadour [29]. The term “Dominance” indicates the proportion (%) of a particular taxon compared to the total number of individuals of all taxa found. Thus, taxa were classified as: “Dominant”: if they constituted 5% or more of the total number of individuals found; “Influent”: if they constituted 2–5%; and “Recedent” if they constituted less than 2%. The “Frequency” of a taxon is measured by the percentage of samples in which the particular taxon was detected. Hence, taxa were classified as: “Constant”: if they were recorded in more than 50% of the total number of samples, “Accessory”: if recorded in 25–50%, and “Accidental”: if recorded in less than 25% of samples. Subsequently, a rank sum test, described as a non-parametric preference data test [55], was performed to assess which of the three different animal baits were more attractive to the captured males of the genus *Sarcophaga*. Only species with five or more males were included in this analysis. Four of the 12 flesh fly species were excluded from this analysis. This threshold was adopted to avoid formal comparisons based on extremely low captures [56]. Following a log transformation that normalized the number of male specimens captured, a one-way Analysis of Variance (ANOVA) was employed to examine the differences between the total attractiveness of species to the three animal baits. ANOVA analysis focused on the two predominant taxa (*S. africa*, *S. melanura*) to determine the effect of each bait on the number of attracted flesh fly specimens. Differences were deemed statistically significant at the level of *p* ≤ 0.05. When ANOVA indicated significance, Tukey’s Honestly Significant Difference Test (Tukey HSD) was employed for post–hoc pairwise comparisons. All ANOVA and Tukey HSD analyses were conducted using Microsoft^TM^ Excel Spreadsheet Software (Microsoft Corporation 2019)and the JMP^®^16 statistical software (SAS Institute 2021, Cary, NC, USA) [57].

## 3. Results

The diversity data presented in this study represent the first faunal composition of flesh flies associated with different types of decomposing baits of animal origin in Greece. A total of 1125 adult flesh fly specimens were collected between May and November from the animal baited traps exposed at the three experimental locations, all representing the genus *Sarcophaga*. Other dipteran and non-dipteran species captured in the baited traps were excluded from the current analyses. Among the captured specimens, 884 were females, which remain unidentified to a species level, while the remaining 241 were males. The female to male average sex ratio was 3.7♀:1♂, with 78.58% of the total captured specimens being females. The study of the males allowed for the taxonomic identification of 12 species (Table 1): *Sarcophaga africa* (Wiedemann), *Sarcophaga argyrostoma* (Robineau–Desvoidy), *Sarcophaga boettcheri* Villeneuve, *Sarcophaga consanguinea* Rondani, *Sarcophaga crassipalpis* Macquart, *Sarcophaga ferox* Villeneuve, *Sarcophaga hirticrus* Pandellé, *Sarcophaga jacobsoni* (Rohdendorf), *Sarcophaga lehmanni* Müller, *Sarcophaga melanura* Meigen, *Sarcophaga minima* Rondani, *Sarcophaga tibialis* Macquart. Accordingly, all subsequent species-level abundance, diversity, dominance, frequency, and bait preference results are based on the identified male specimens.

The overall species composition analysis revealed that *S. africa* was by far the most common taxon, with a total of 100 specimens (representing 41.50% of the total capture) collected throughout the entire sampling period of the experiment. The second most abundant species was *S. melanura* (20.75%), followed by *S. tibialis* and *S. hirticrus* with 8.71% each, and finally *S. jacobsoni* (6.22%) and *S. ferox* (5.39%). All remaining taxa (*S. consanguinea*, *S. minima*, *S. boettcheri*, *S. argyrostoma*, *S. crassipalpis*, *S. lehmanni*) constituted the rest of the sarcophagid community (8.71%). Among all baits, beef liver trapped the highest number of male flesh fly specimens (*n* = 89), followed by fish (*n* = 82) and finally chicken stomach (*n* = 70). Baits composed of a single animal tissue were unable to attract all the different species (Table 1). Beef liver and fish baits demonstrated the highest species diversity, with beef liver bait capturing 11 out of the total 12 species collected over the sampling period, and fish bait capturing 10 out of the total 12 species collected. Notably, single specimens of *S. argyrostoma* and *S. consanguinea* were present only on beef liver and fish baits, respectively (Table 1). In contrast, chicken stomach bait exhibited the lowest species diversity, capturing 8 out of the 12 species during the study period. The occurrence of all male species varied across the sampling months.

As evident from Table 2, species such as *S. africa* and *S. ferox* were present in all sampling months, except in November, whereas *S. hirticrus*, *S. melanura*, and *S. tibialis* were observed in all samples except those collected in November and September. Species including *S. boettcheri*, *S. lehmanni*, *S. minima*, and *S. crassipalpis* were detected only in June and July, with *S. crassipalpis* also appearing in September. *Sarcophaga jacobsoni* was recorded in all months except June and August, while *S. consanguinea* was detected only in October and *S. argyrostoma* only in July.

The results revealed that six species were classified as dominant (*S. africa*, *S. melanura*, *S. hirticrus*, *S. tibialis*, *S. jacobsoni*, *S. ferox*). The two predominant taxa, *S. africa* and *S. melanura*, maintained a constant presence throughout the sampling period, while the four other dominant taxa (*S. hirticrus*, *S. tibialis*, *S. jacobsoni*, *S. ferox*) were all categorized as accessory. The remaining 8.71% of the collected specimens encompassed six species (*S. minima*, *S. boettcheri*, *S. consanguinea*, *S. argyrostoma*, *S. crassipalpis*, *S. lehmanni*), all reported as recent, except for *S. minima* and *S. boettcheri*, which were classified as influent in terms of their dominance, whereas all six species were classified as accidental in terms of their frequency (Table 3).

### Preferences for Type of Bait

Using the non-parametric preference data test [55], only 232 male specimens of *Sarcophaga* spp. were included. The results revealed that 36.21% of the *Sarcophaga* spp. were captured in traps baited with beef liver, 29.74% in traps baited with chicken stomach, and 34.05% in traps baited with fish. The remaining eight species of *Sarcophaga* spp. were analyzed separately. *Sarcophaga africa* preferred beef liver bait, three species (*S. ferox*, *S. minima*, *S. boettcheri*) preferred chicken stomach bait, and another three species (*S. hirticrus*, *S. jacobsoni*, *S. tibialis*) preferred fish bait. Species *S. melanura* preferred chicken stomach and fish baits equivocally, but exhibited no preference towards beef liver bait (Table 4).

The two predominant taxa (*S. africa* and *S. melanura*) were further analyzed individually. The bait preferences for the species *S. africa*, when analyzed using an ANOVA, revealed that there were statistical differences between the three bait types (df = 2, 26, F = 4.15, *p* < 0.05). Post hoc pairwise Tukey HSD comparisons between baits showed that beef liver was significantly different from fish bait (*p* < 0.05) but not significantly different from chicken stomach bait (*p* > 0.05). Additionally, no differences were detected between chicken stomach and fish baits (*p* > 0.05). Overall, using an ANOVA, there was no significant difference between baits attracting *S. melanura* (*p* > 0.05).

An overview of the daily data from the weather station was compiled, and the averages for temperature and relative humidity were calculated. Sampling months and a summary of the average temperature (T_mean_) (°C) and average relative humidity (RH_mean_) (%) during each sampling month are presented in Figure 3. The month of November was excluded from the environmental summary because sampling was limited to a single event.

Overall, the sampling period was characterized by warmer and relatively drier summer conditions followed by cooler and more humid early autumn conditions. Mean temperature increased from approximately 24 °C in May to about 30–31 °C in July and August, before declining to around 25 °C in September and 19 °C in October. Mean relative humidity fluctuated between approximately 44% and 54% from May to August, before increasing to about 61–62% in September and October.

## 4. Discussion

The present study is the first to report on both the diversity and abundance of Sarcophagidae species attracted to three types of decomposing protein baits in Athens, Greece. The use of carrion-baited traps is common practice in the study of sarcosaprophagous Diptera [16,25,27,58,59,60,61,62,63,64]. The use of decomposing baits, such as animal tissues, to attract and collect Sarcophaginae is a good sampling strategy. Necrophagous and omnivorous fly species seek these types of resources to address their dietary and reproductive processes for larval growth and ovarian development, or to select a mating site [65]. Interestingly, among the Sarcophagidae collected in this study, only species of the genus *Sarcophaga* were attracted to the baited traps. A study conducted by Villet et al. [27] in South Africa using chicken-baited traps also trapped species exclusively belonging to the genus *Sarcophaga*. This was not observed by other authors using traps baited with other animal tissues, who instead reported a variety of flesh fly species being attracted [64,66,67]. One explanation for this is that this genus has many facultative behaviors (coprophagy, necrophagy, saprophagy, predation or parasitism) [68], as well as being very competitive carrion visitors [69], many of forensic importance [70,71]. An alternative reason may be that *Sarcophaga* species were just more prevalent in the study area during the sampling period, reflecting their dominance in the local fly community. Pekbey [68] also recorded many flesh flies during the summer months in Türkiye, which is commensurate with their activity period in the Mediterranean region, from spring to mid-autumn [25].

The present study identified twelve species of Sarcophagidae. This discovery is particularly significant as the existing knowledge of the diversity, distribution, and ecological patterns of flesh flies in Greece is limited [37,38]. Of the twelve identified taxa, six were determined to be the most abundant. Hanski [15] has previously shown that carrion-associated insect assemblages are generally dominated by a single or a few dipteran species. In this study, *S. africa* was the most abundant taxon, followed by *S. melanura*. This observation is consistent with the literature, which notes that these two species, among others, such as *S. argyrostoma*, *S. caerulescens*, *S. dux*, and *S. similis*, dominate in decomposing animal tissue in Europe [2]. In a similar study in South Africa, *S. africa* dominated the sample throughout the entire sampling period [27]. *Sarcophaga africa* was found to be the most generalist species in other similar studies [27,64]. Thus, the habitat preference and habits known for both species are consistent with our results. However, adult occurrence and abundance on carrion does not necessarily indicate confirmed larval development on carrion, particularly as *S. africa* larvae develop in a range of organic substrates and show a distinct preference for feces, although they have also been reported from animal carrion [72,73,74]. The remaining four species, in order of abundance, were *S. tibialis*, *S. hirticrus*, *S. jacobsoni*, and *S. ferox*, which have also been reported occasionally in decomposing tissue [2,31,75,76]. The overall flesh fly species composition collected during this study reflects many of the same species recorded visiting carrion, baited traps, or human remains in other regions [2,27,31,46,71,75,76,77,78]. In the present study, even species not trapped in high numbers, such as *S. argyrostoma* and *S. crassipalpis*, are recognized as cosmopolitan and synanthropic species frequently collected from decomposing tissue in similar environments [11,79,80]. The low abundance and frequency (only single specimens captured) of species like *S. consanguinea* and *S. argyrostoma* highlight the potential for site-specific factors influencing species presence. What is known is that *S. argyrostoma* has necrophagous preferences, and it is well known as a regular colonizer of large carrion in European anthropogenic habitats, including being found indoors, particularly in urban areas [81]. Also, the larvae of *S. consanguinea* are known to develop as parasitoids of the terrestrial snail *Theba pisana* (O. F. Müller) [37,82]. Another species caught in low numbers and associated with rotting animal tissue was *S. lehmanni*, which was only recorded in traps placed near the lairage facilities, and only during the months of June and July, consistent with literature reporting that adults of this species are attracted to meat and feces [83] and prefer warm, sunlit habitats [37]. Nevertheless, the presence of adults on carrion should not be interpreted as evidence that carrion serves as a natural breeding substrate for this particular species, as there are no taxonomically verified records of larval development in carrion under natural conditions.

The variation in attractiveness to different animal baits is generally attributed to the specific odors they emit [24], and it also depends on the physiological state of the flies, such as sex, age, nutritional and reproductive status [84]. Similarly to this study, Sánchez–Rojas et al. [85] noted that by deploying a variety of different baits (fresh to aged), a broader range of fly species may be captured [32], demonstrating these differences in bait preferences.

The current study showed that baits alone and in combination demonstrated variation in the effectiveness of attracting adult flies of the family Sarcophagidae. Due to beef liver attracting a high number of Sarcophagidae species with a high species diversity (eleven out of the twelve identified species) (Table 1), the effectiveness of this bait may be useful for capturing this group of flesh flies in similar Mediterranean regions. Fish bait also showed attractiveness to ten species, while chicken stomach attracted the least diversity, with only eight species. Other similar studies have shown that beef liver is an effective bait in terms of attracting necrophagous flies [4,29,63,86]. This may be attributed to its high nutrient and protein content [87]. These findings are critical for optimizing bait selection in local investigations to ensure the capture of a representative sample of the sarcophagid population.

Furthermore, both pitfall and bottle traps captured a diverse range of the identified species, indicating both are viable collection methods for sarcosaprophagous species surveys. As evident from Table 2, apart from the species recorded only once (*S. argyrostoma*, *S. consanguinea*), all identified male species were collected using both types of traps. This indicates that both trap types are effective collection methods for capturing sarcophagid species. In this current study, distinct patterns of attraction to the three baits by the different flesh fly species were recorded. Specifically, *S. africa* showed an attraction towards beef liver, while *S. hirticrus*, *S. jacobsoni*, and *S. tibialis* were attracted to fish. In contrast, *S. ferox*, *S. minima*, and *S. boettcheri* showed an attraction to chicken stomach bait. Subsequent comparisons based on *S. africa* and *S. melanura* revealed that *S. africa* preferred beef liver over fish bait and showed no significant preference between beef liver and chicken stomach bait. Similar studies have demonstrated the attraction of *S. africa* to beef liver and/or chicken baits [27,40,65].

The attractiveness of *S. africa* to beef liver compared with fish bait suggests that this species may respond differently to particular decomposing animal tissues under urban Mediterranean conditions. This finding is biologically relevant because it points to possible differences in carrion resource selection among sarcophagid species. It is also important methodologically, as beef liver appeared to be an effective bait for detecting *S. africa*, the predominant species in the present study. However, this does not imply that beef liver is equally effective for all sarcophagids; rather, it emphasizes that bait type can influence the richness and composition of necrophagous fly assemblages [64]. From a forensic perspective, this finding can help guide future surveys in Greece and similar Mediterranean environments by identifying bait types useful for documenting locally important flesh fly species [37]. This is particularly relevant for species such as *S. africa*, which has confirmed forensic relevance in Europe [2,12,16,37,71]. On the other hand, *S. melanura* preferred all three baits equally. *Sarcophaga melanura* seems to be more diverse in its choice of substrates, as it has been reported on animal remains, fecal substrates, and sometimes may predate other fly larvae [51,53,88].

In the present study, sarcophagid species varied across the sampling months, with occurrences concentrated mainly in the warmer months (Table 2, Figure 3). This pattern is consistent with Martín–Vega and Baz [25], who found seasonal differences in sarcosaprophagous Diptera assemblages and reported that most sarcophagid species were more abundant during summer. Furthermore, the current study shows that temperature and humidity are likely factors in attracting both female flies and the total number of adult flies to the baits. This is partly supported by De Souza and Von Zuben [63] in Brazil, who used minced meat, sardines, and beef liver as baits, whereby significantly more Sarcophagidae specimens were collected during the warmer months.

Laboratory observations show that the sex ratio of emerged individuals is approximately 1:1 [89,90]. However, the observed female biased sex ratio of 3.7♀:1♂ in this study is most likely attributable to females seeking protein sources to become sexually receptive and develop their eggs/larvae [91,92,93]. In contrast, males generally prioritize their carbohydrate intake to meet their high caloric requirements for mobility, and often guard protein sources to encounter and copulate with mature females [22]. Additionally, the more developed olfactory receptors in female necrophagous flies, as indicated by the greater number of sensory pits in the antenna sensilla [94,95], may enhance their attraction to carrion [96,97]. As well, monitoring the abundance and diversity of sarcosaprophagous Diptera using carrion-baited traps can be beneficial for investigating their population dynamics and evaluating various strategies for controlling animal myiasis [98].

Within the broader Mediterranean context, the present findings show considerable overlap with a recent study by Bonelli et al. [99] in Calabria, southern Italy. Nine of the twelve *Sarcophaga* species (*S. africa*, *S. melanura*, *S. tibialis*, *S. hirticrus*, *S. jacobsoni*, *S. argyrostoma*, *S. crassipalpis*, *S. consanguinea*, and *S. lehmanni*) identified in the present study were also recorded in Calabria. Despite this overlap in species composition, the relative abundance patterns differed between the two studies. In Athens, *S. africa* and *S. melanura* were the predominant species, whereas Bonelli et al. [99] recorded *S. croatica* as the dominant sarcophagid, with *S. africa* and *S. melanura* occurring at lower relative abundances. The authors also associated *S. africa* with open habitats and reported associations of *S. africa*, *S. argyrostoma*, and *S. melanura* with temperature and human activity, providing an ecological parallel with their occurrence in the urban Mediterranean environment of Athens. Differences in community structure between the two studies may also reflect differences in habitat coverage, altitude, sampling duration, and bait methodology. Nonetheless, there is similarity in the sarcophagid fauna between Athens and southern Italy, but contrasting dominance patterns exist, which may reflect the importance of local ecological conditions in shaping flesh fly assemblages.

Several considerations should be taken into account when interpreting the present findings. First, the current study was conducted within a single urban area of Athens (Figure 1) and therefore may not fully represent sarcophagid communities in other regions of Greece or in rural, coastal, mountainous, or less anthropogenic habitats [37]. In addition, sampling was limited to one study period, and the observed species composition may have been influenced by seasonal conditions during the months sampled (Figure 3), as sarcosaprophagous Diptera assemblages can vary spatially and seasonally [25]. Another important consideration is that female specimens were identified only to genus level, because reliable species-level identification in members of the genus *Sarcophaga* is often challenging using morphology-based approaches due to similarities among females [2,12,71]. As a result, the species level analyses represent the identified male component of the assemblage. Combining morphological identification with molecular tools could also help overcome some of the difficulties associated with species level identification, including cases where female specimens cannot be reliably identified morphologically [100]. Furthermore, wing measurements have the potential to be particularly advantageous in the Sarcophagidae because they are equally efficient in the identification of both sexes [71].

Understanding the insect fauna in specific geographic locations constitutes a fundamental step in applying entomological knowledge to civil and criminal cases. FE still remains an underdeveloped discipline in Greece [29], unlike many other countries worldwide where it is routinely conducted [101]. The current local knowledge in Greece concerning the family Sarcophagidae is increasing [37,38]. By providing a clearer understanding of the species present in the Athens local area, it facilitates the design of future research initiatives to address many of the considerations identified above.

## 5. Conclusions

This study represents the first documentation of Sarcophagidae species diversity in Athens, Greece, utilizing carrion-baited traps to evaluate species richness and bait attractiveness. A total of 12 *Sarcophaga* spp. were identified. Beef liver emerged as the most effective bait, capturing the highest diversity and number of specimens, followed by fish and chicken stomach, although no single bait attracted all recorded species, highlighting that, in combination, a much better understanding of this diversity was achieved. The research underscores the importance of localized entomological studies in enhancing forensic practices and sets a baseline for further investigations into the ecology and forensic relevance of Sarcophagidae across Greece. Future studies should expand sampling to additional regions and habitat types, examine seasonal and ecological patterns, and incorporate molecular and geometric morphometric tools to improve species level identification. This information may support the interpretation of sarcophagid evidence recovered from decomposing remains, especially when assessing whether collected insects are consistent with the fauna, habitat conditions, and environmental context of a case.

## Figures and Tables

**Figure 1:**
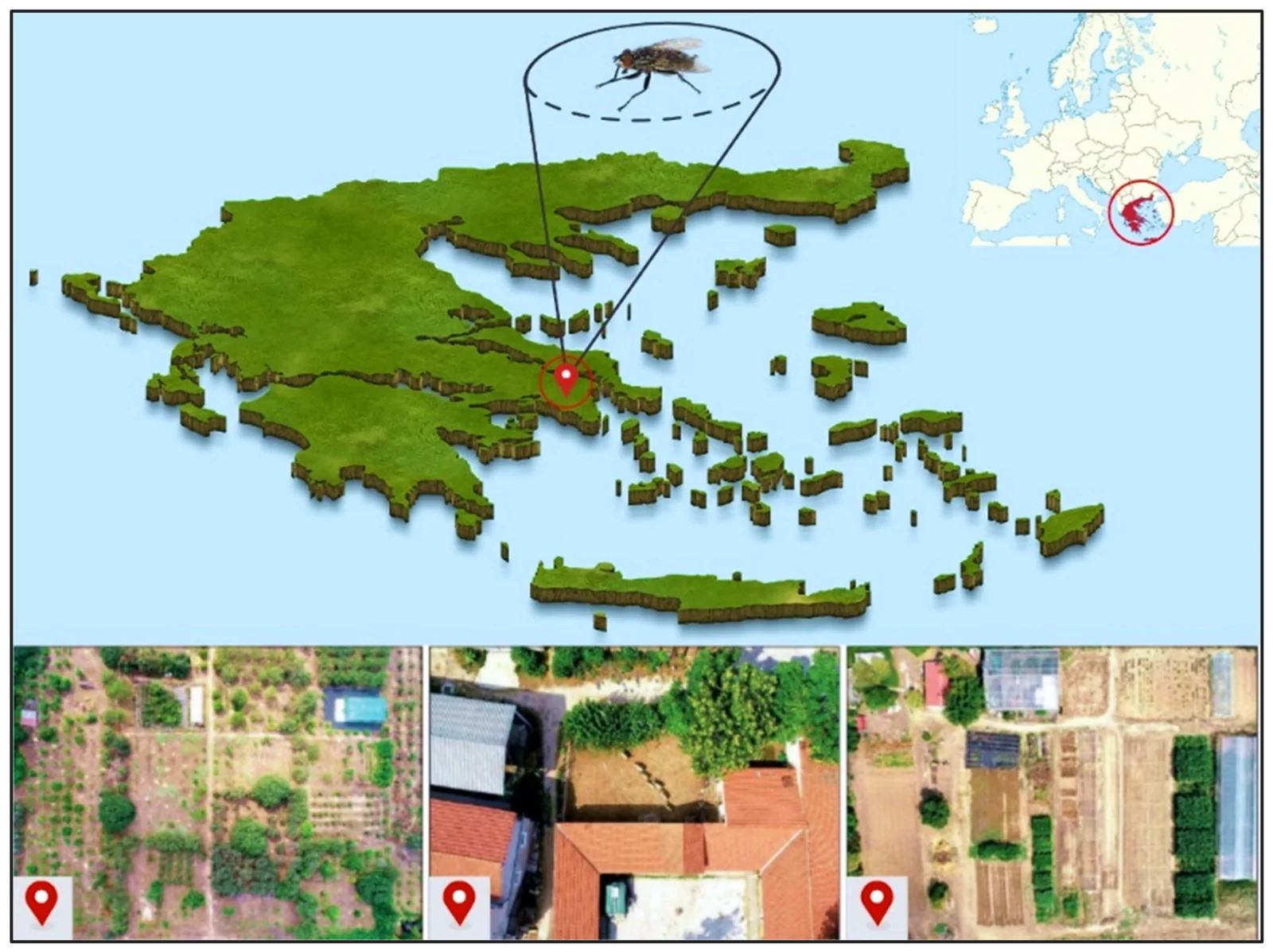
Depiction of the Athens greater urban area. The red location pins (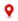) denote the municipality of Athens, Attiki, Greece, where the study was conducted, as well as the collection sites. Bottom row: Aerial views of the three collection sites on the grounds of the AUA (from left to right): Experimental orchard; Experimental livestock facilities; Arable crops area.

**Figure 2:**
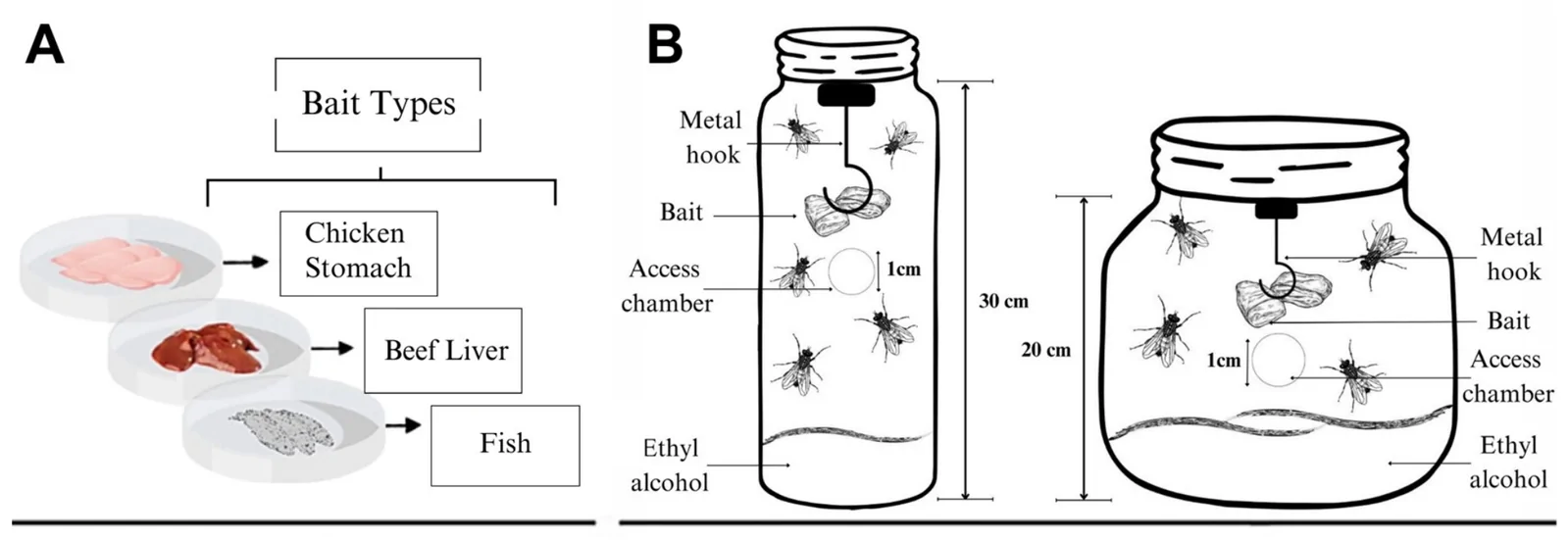
Sampling design used in the study. (**A**) Bait types used for specimen collection: chicken stomach, beef liver, and fish. (**B**) Schematic diagrams of the two baited trap types used for sampling: bottle trap (**left**) and pitfall trap (**right**).

**Figure 3:**
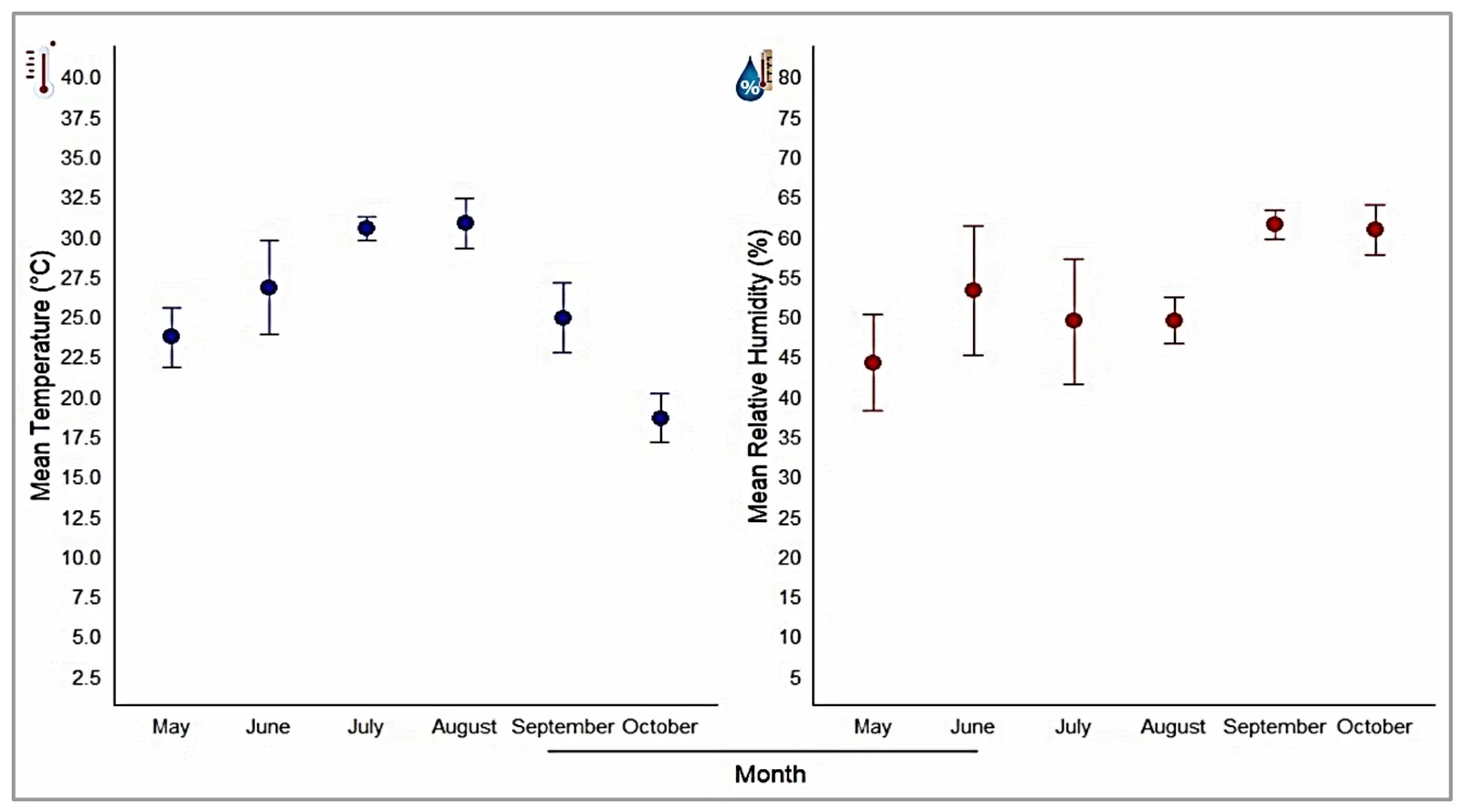
Weather data. Average temperature and relative humidity variation across the sampling period.

**Table 1:** Abundance and occurrence of *Sarcophaga* spp. (males only) captured by type of bait during the sampling period.

Species ID	Chicken Stomach	Beef Liver	Fish	Total
*Sarcophaga* (*Bercaea*) *africa*	28	52	20	100
*Sarcophaga* (*Helicophagella*) *hirticrus*	5	5	11	21
*Sarcophaga* (*Helicophagella*) *melanura*	18	14	18	50
*Sarcophaga* (*Heteronychia*) *boettcheri*	3	2	2	7
*Sarcophaga* (*Heteronychia*) *consanguinea*	0	0	1	1
*Sarcophaga* (*Heteronychia*) *ferox*	6	3	4	13
*Sarcophaga* (*Heteronychia*) *minima*	3	1	1	5
*Sarcophaga* (*Liopygia*) *argyrostoma*	0	1	0	1
*Sarcophaga* (*Liopygia*) *crassipalpis*	0	1	2	3
*Sarcophaga* (*Liosarcophaga*) *jacobsoni*	0	4	11	15
*Sarcophaga* (*Liosarcophaga*) *tibialis*	6	3	12	21
*Sarcophaga* (*Sarcophaga*) *lehmanni*	1	3	0	4
Total per bait type	70	89	82	241

**Table 2:** A summary of the sampling data: Species collected, total number of collected specimens, location of collection, collection month, and trap type. Acronym legend: In locality (corresponds to sites in study), (OLA—Orchard, Livestock Facilities, Arable Crops; O—Orchard, OA—Orchard, Arable Crops, A—Arable Crops, LA—Livestock Facilities, Arable Crops, L—Livestock Facilities); In Collection Month(s) (corresponds to months of study), (Jun = June, Ju = July, Aug = August, Sept = September, Oct = October, Nov = November); In Τrap Τype (corresponds to trapping method used in study), (B = bottle trap, P = pitfall trap).

Species	No. of Individuals	Locality	Collection Month(s)	Trap Type
*S. africa*	100	OLA	May–Oct	B, P
*S. hirticrus*	21	OLA	May–Aug, Oct	B, P
*S. melanura*	50	OLA	May–Aug, Oct	B, P
*S. consanguinea*	1	O	Oct	P
*S. ferox*	13	OLA	May–Oct	B, P
*S. minima*	5	OA	Jun, Jul	B, P
*S. boettcheri*	7	OA	Jun, Jul	B, P
*S. argyrostoma*	1	A	Jul	B
*S. crassipalpis*	3	LA	Jun, Jul, Sept	B, P
*S. jacobsoni*	15	OLA	May, Jul, Sept–Nov	B, P
*S. tibialis*	21	OLA	May–Aug, Oct	B, P
*S. lehmanni*	4	L	Jun, Jul	B, P

**Table 3:** Dominance and frequency of identified male flesh fly species. [Dominance: –Dominant (>5%); –Influent (2–5%); –Recedent (<2%), Frequency: –Constant (>50%); –Accessory (25–50%); –Accidental (<25%)].

Species	% of the Total No. of Specimens	Dominance	% of the Total No. of Samplings	Frequency
*S. africa*	41.50	Dominant	92.59	Constant
*S. hirticrus*	8.71	Dominant	44.44	Accessory
*S. melanura*	20.75	Dominant	59.26	Constant
*S. consanguinea*	0.41	Recedent	3.70	Accidental
*S. ferox*	5.39	Dominant	33.33	Accessory
*S. minima*	2.07	Influent	11.11	Accidental
*S. boettcheri*	2.90	Influent	14.81	Accidental
*S. argyrostoma*	0.41	Recedent	3.70	Accidental
*S. crassipalpis*	1.24	Recedent	11.11	Accidental
*S. jacobsoni*	6.22	Dominant	25.93	Accessory
*S. tibialis*	8.71	Dominant	37.04	Accessory
*S. lehmanni*	1.66	Recedent	11.11	Accidental

**Table 4:** Bait preferences of combined (*Sarcophaga* spp.) and individual flesh fly species.

Taxa	Bait Type		
	Chicken Stomach	Beef Liver	Fish
*S. africa*	28.00%	52.00%	20.00%
*S. hirticrus*	23.81%	23.81%	52.38%
*S. melanura*	36.00%	28.00%	36.00%
*S. ferox*	46.15%	23.08%	30.77%
*S. minima*	60.00%	20.00%	20.00%
*S. boettcheri*	42.86%	28.57%	28.57%
*S. jacobsoni*	0.00%	26.67%	73.33%
*S. tibialis*	28.57%	14.29%	57.14%
Total analyzed *Sarcophaga* spp. (*n* = 232)	29.74%	36.21%	34.05%

## Data Availability

The flesh fly specimens listed in this study are deposited in the personal entomological collection of the first author and the KMNH and are available from the curators upon request. The datasets generated during this study are available from the corresponding author upon request. Further inquiries can be directed to the corresponding author [G.D.R.].
